# Advanced 3D Facial Scanning in Orthodontics: A Correlative Analysis of Craniofacial Anthropometric Parameters

**DOI:** 10.3390/jcm14217578

**Published:** 2025-10-25

**Authors:** Andra-Alexandra Stăncioiu, Alexandru Cătălin Motofelea, Adelina Popa, Riham Nagib, Rareș-Bogdan Lung, Camelia Szuhanek

**Affiliations:** 1 Orthodontic Research Center ORTHO-CENTER, Discipline of Orthodontics I, Faculty of Dental Medicine, “Victor Babes” University of Medicine and Pharmacy Timisoara, 9 No., Revolutiei Bv., 300041 Timisoara, Romania; andra.stancioiu@umft.ro (A.-A.S.); popa.adelina@umft.ro (A.P.); nagib.riham@umft.ro (R.N.); rares-bogdan.lung@student.umft.ro (R.-B.L.); cameliaszuhanek@umft.ro (C.S.); 2 Center for Molecular Research in Nephrology and Vascular Disease, Discipline of Nephrology, Department VII/Internal Medicine II, Faculty of Medicine, “Victor Babes” University of Medicine and Pharmacy, 300041 Timisoara, Romania

**Keywords:** dentistry, facial morphometry, measurements, MetiSmile Shining 3D Facial Scanner, soft tissues

## Abstract

**Background/Objectives:** This study aims to investigate the correlation between vertical facial dimensions, lip morphology, angular facial parameters, and demographic variables (age and sex) in Romanian orthodontic patients, using structured-light 3D facial scanning technology. **Methods:** This cross-sectional study assessed craniofacial soft tissue parameters in 90 Romanian orthodontic patients (57 females, 33 males; median age 14 years) using the MetiSmile 3D facial scanner. Measurements included vertical facial heights (upper, middle, and lower), total facial height, facial proportion indices, lip lengths, interpupillary distance, bizygomatic width, mouth width, angular parameters (nasofrontal, nasolabial, mentolabial, and facial angles), and distances from the lips to the esthetic (E) line. Spearman’s rank correlation and Mann–Whitney U-tests were applied for statistical analysis. **Results:** A total of 90 subjects (63.3% female; median age, 14 years [range: 9–27 years]) were evaluated. Age showed strong positive correlations with total facial height (ρ = 0.690), middle (ρ = 0.631), and lower facial height (ρ = 0.615), while upper facial height had a weaker correlation (ρ = 0.334). Upper and lower lip lengths were moderately correlated with each other and with bizygomatic and interpupillary widths. Vertical facial proportion indices reflected distinct associations with their respective facial segments. Sex-based analysis revealed that females had significantly greater middle and total facial heights and nasofrontal angles, while males exhibited larger nasolabial angles. Upper lip protrusion relative to the E-line was significantly more pronounced in females (*p* = 0.041). No significant sex differences were noted in lip dimensions or transverse widths. **Conclusions:** The research demonstrates the value of 3D structured-light facial scanning in orthodontics as a non-invasive, radiation-free method for evaluating age- and sex-related morphological patterns. These findings support the integration of facial morphometric analysis into individualized diagnostic and treatment workflows in clinical orthodontics.

## 1. Introduction

Three-dimensional (3D) facial scanning has transformed clinical dentistry, offering precise digital representations of facial features for improved diagnosis, treatment planning, and outcome assessment. Compared to manual methods and 2D photography, 3D scanning allows for objective analysis of facial morphology—crucial in orthodontics, prosthodontics, and maxillofacial surgery. It also enhances patient communication and planning accuracy, while recent advances in portability and software integration have increased its clinical accessibility [1].

Advancements in digital technology have significantly enhanced dental procedures. While intraoral scanners and CAD/CAM systems are now standard, facial scanners have recently become more integrated into digital workflows. These devices capture detailed 3D images of the head and face for use in diagnosis, treatment planning, and communication. Although their use has grown in recent years, facial scanning in dentistry dates back to 1991, when Moss et al. first applied 3D laser scanning in a clinical setting [2].

Pretreatment and ongoing clinical data collection and analysis are made easier by facial scanning technologies. Combining image files from a facial scanner, intraoral scanner, and CBCT to create a virtual patient can expedite diagnostic processes, enable precise patient analysis, and function as an effective communication tool to convey information to the lab technician and simulate treatment outcomes for the patient. Facial scanning can also be applied in aesthetic dentistry as a crucial step in the process of creating a digital smile [3]. In addition, a virtual teeth setup with a 3D model can be used to digitally assess tooth positions, shapes, and colors on the face in immediate dentures by using a facial scanner to record natural rest position, maximum smile, and various grin lines [4]. Dental technology has advanced thanks to digital technology and facial scanners, but their novelty and expense have mostly prevented them from becoming widely used. Gaining an understanding of how facial scanning can be integrated into dental procedures will help professionals feel more at ease using this technology [2].

It has been established that facial scanning accuracy is clinically suitable for usage in dentistry applications. By comparing the scan to a control model and calculating the differences between the two, accuracy is determined. It is deemed acceptable for scanners to have deviation values below 2 mm [5]. Facial scanning deviation values fall between 140 and 1330 μm. The majority of facial scanners had reported accuracy of about 500 μm, which is within clinically acceptable bounds. However, a number of variables, like the type of scanner being used, the geometry of the object, and the depth and speed of scanning, might affect scanner accuracy [2]. The shape of the object affects the facial scan’s accuracy as well. Research has indicated that the lower face’s concave surfaces were less accurate than the top face’s flat and convex surfaces [6]. The labiofacial sulcus, oral commissures, and oral fissures were among the areas with undercuts that had worse scanning accuracy and higher scanning difficulty. In contrast to the top and lower faces, the midface exhibited the highest level of precision and overall accuracy performance. The extra-auditory canal, nostrils, and teeth were additional features that were more challenging to photograph [7]. Accuracy is also influenced by scanning speed and depth. Accuracy was worse for measurements of regions deeper than 2 mm. A failure to pass light into deep places during scanning could be the cause of this error. It was demonstrated that closer scanning methods produced more accurate scans. The accuracy of the scan declined with increasing scanning length [2].

Accurate treatment planning requires comprehensive patient records. Facial scanners can digitize and replace traditional tools like facebows and wax-ups, streamlining communication between clinicians, labs, and patients. When combined with intraoral scans, CBCT, and digital photography, facial scans allow for “best fit” analysis to align datasets and create a precise 3D virtual patient model [8].

Soft tissue-driven concerns are currently driving a paradigm change in orthodontic treatment planning. Despite the fact that 3D facial diagnosis is unquestionably important for treatment planning, it has proven challenging to capture using conventional 2D digital diagnostic methods. Although the cost of high-end facial scanners is delaying this shift, the 3D diagnostic workflow will soon be accepted as standard practice [9]. Dental imaging is a common clinical practice that provides valuable information for a number of uses. In various specialties, including orthodontics, orthognathic surgery, and facial plastic surgery, soft tissue photographs of the face are crucial documentation for assessing the maxillofacial region. These images are utilized for a variety of purposes, including outcome analysis, treatment planning, and diagnostic processing [9].

When combined with a digital study model and a CBCT scan, facial scanners can provide a 3D topography of a patient’s facial surface anatomy, creating a 3D “virtual patient” that can help with diagnosis, treatment planning, and patient outcomes [9].

Facial recognition systems, a branch of AI, use deep learning to identify individuals by analyzing facial features such as distances between landmarks or skin texture. Trained on large datasets, these systems create facial profiles for comparison. According to NIST, top algorithms have improved rapidly, with error rates dropping from 4.1% in 2014 to just 0.08% in 2020 [10].

Since the change in body mass index is not a particularly representative value when the facial morphology is the metric, facial scanning can provide helpful correlative data for many studies that would benefit from routine, noninvasive evaluations of head and neck soft-tissue morphology [9].

Dental face scanners are increasingly used for accurate diagnosis, treatment planning, and outcome assessment. These extraoral devices capture high-resolution 3D facial images using structured light or laser technology. The data can be integrated with intraoral scans and CBCT for advanced digital workflows, improving efficiency, communication, and treatment precision. Key factors when evaluating scanners include accuracy, resolution, scan time, and ease of use—crucial for aligning facial and intraoral data in aesthetic and functional treatments [11].

Recent advancements in automated landmark recognition utilizing deep-learning frameworks have facilitated swift and repeatable 3D face morphometry, diminishing operator reliance and enhancing diagnostic efficiency [12].

Furthermore, research affirming mobile-based structured-light or TrueDepth-camera scanning devices for face anthropometry underscores the increasing accessibility of digital processes in orthodontics [13].

These results underscore the need for population-specific normative data and ethnicity-conscious reference models in 3D face scanning research.

Recent improvements in automated 3D facial landmark detection and software-based morphometric techniques have enabled high-throughput, consistent facial measurements across various populations [14].

Concurrent studies have underscored the significance of ethnic and demographic diversity in 3D face anthropometry, revealing substantial disparities based on race/ethnicity, age, and sex [15,16].

These findings support the need for population-specific reference models in digital orthodontic research.

The aim of this study was to introduce 3D facial scanning as a modern, non-invasive tool for orthodontic assessment. We investigated correlations between key facial anthropometric parameters, including vertical and proportional facial heights, lip lengths, transverse facial dimensions, soft tissue angles, and E-line-related measurements, using standardized 3D orthodontic morphometry.

## 2. Materials and Methods

The research focused on patients who were enrolled in the Orthodontics I Discipline at the Faculty of Dental Medicine, “Victor Babes” University of Medicine and Pharmacy, Timisoara. Every participant gave written informed consent, and the study was accepted by the “Victor Babes” University of Medicine and Pharmacy’s Institutional Ethics Committee in Timisoara, Romania (CECS Nr. 04/26 January 2024).

This study focused on the correlation between facial anthropometric parameters, or facial morphometry, by performing facial anthropometric measurements using a facial scanner. For the sample, a minimum of 90 patients was needed. The effect size was based on a previous study [11].

The study included 90 Romanian patients who sought orthodontic treatment at the university clinic to enhance their facial and dental features and achieve a functional and stable occlusion. Every patient underwent a comprehensive clinical evaluation that included radiography such as OPG and posterior–anterior and lateral cephalograms, facial and dental scanning, pre-treatment 3D-printed study models, and clinical photographs (intraoral and extraoral). Criteria for Inclusion: those slated for or already receiving orthodontic treatment. Age range: 14 (10–24). High-quality 3D facial scans, obtained through structured light, are readily available. The MetiSmile facial scanner, developed by Shining 3D, is an example of a structured light 3D scanner. Structured light scanners employ cameras to record the way a known pattern—usually stripes or grids—deforms across the subject’s face after projecting it onto it. The 3D geometry of the face is highly accurately calculated using this deformation. There should be no significant facial injuries or previous orthognathic surgery on the subject. The ability to observe one’s face with a neutral expression is crucial. The MetiSmile FacialPlan program executes automatic landmark identification and linear/angular measuring with structured-light 3D data. Calibration accuracy is guaranteed by the use of the manufacturer’s reference board and internal algorithm validation, attaining sub-millimeter measurement precision in accordance with the device requirements. Criteria for exclusion were as follows: individuals with congenital facial abnormalities or craniofacial syndromes (such as Treacher Collins syndrome or cleft lip/palate); a history of facial reconstructive or cosmetic surgery unrelated to orthodontics; the presence of facial hair or accessories, like spectacles or body piercings, which can impact the quality of the scan, as the 3D facial scan may contain insufficient or low-quality information; uncooperative actions (such as movement or overly dramatic facial expressions) while scanning.

### 2.1. Procedure Methodology

All scans were conducted by a single trained operator according to the manufacturer’s prescribed calibration process. The ambient illumination was regulated to eliminate shadows and glare, while the scanning configuration (distance, orientation, and exposure) remained the same for all participants. Patients were advised to tie back any hair that was blocking their forehead or other facial landmarks and to take off any facial accessories, such as glasses, earrings, or facial piercings, before scanning. To reduce shadows and reflections, the scanning process was carried out in a regulated setting with uniform lighting. On a sturdy, height-adjustable chair, patients were seated upright. They were positioned in accordance with the manufacturer’s suggested scanning range, which is normally 35 to 60 cm, from the MetiSmile facial scanner. To provide uniform head orientation, the Frankfort horizontal plane was positioned parallel to the ground. The subjects were instructed to look at a fixed location on the wall in front of them at eye level in order to preserve a natural head position (NHP). They were told not to strain or smile and to keep their faces neutral and relaxed, with their lips slightly closed and their teeth in maximal intercuspation (centric occlusion). The operator started the scanning procedure after the patient was properly positioned. In just a few seconds, the 3D facial image was acquired by the MetiSmile scanner using structured light technology. To guarantee accuracy and completeness, the scan was examined in real time. The scan was repeated in the event of any notable face movement or data loss [17,18]. The natural head position (NHP), which involves holding the head up straight and focusing the eyes at eye level on a faraway object, was developed to achieve the proper head position [19].

For this study, we used the MetiSmile Shining 3D Facial Scanner (Shining 3D, China), the first facial scanner developed specifically for dentistry. Using advanced algorithms, it records multiple facial perspectives in under 10 s to create a μm-level accuracy 3D model. The device incorporates three data acquisition cameras and a 5.0 MP HD texture camera for high-fidelity textures and realistic color, and can automatically align facial scans with intraoral data for orthodontic “before/after” simulations. The Ortho Simulation module enables one-click tooth segmentation and lip extraction, while an optional Mandibular Trajectory Tracking module captures dynamic occlusal data. The scanner is compact (215 × 50 × 75 mm), lightweight (800 g), portable, and outputs STL, OBJ, and PLY files. It operates using infrared, flash-free scanning with automatic brightness adjustment. Technical specifications include FOV 210 × 270 mm at 500 mm working distance, LED color temperature 5500 K, input AC 100–220 V, 50/60 Hz, 1.5 A, and output DC 12 V, 7.0 V. Recommended computer configuration is as follows: Intel Core i7-8700+, 32 GB RAM (minimum 16 GB), ≥256 GB SSD, Nvidia RTX 2060 6 GB+, monitor resolution ≥ 1920 × 1080 at 60 Hz, Windows 10/11 64-bit, USB 3.0 [20].

In this study, we used a Lenovo Legion 5 Pro 16ARH7H laptop (AMD Ryzen™ 7 6800H, 16″ WQXGA IPS, 16 GB RAM, 512 GB SSD, NVIDIA GeForce RTX 3070 8 GB) to run the Shining 3D FScan software (v. 2.2.0.4) for facial scan data acquisition. An institutional account was created, and new scans were initiated via the “New Order” function by entering patient and clinical details (name, clinician, visit type, and treatment category). Scans were performed using the “Scan” command, followed by “Pre-design” and “Send” to save the data. Measurements were conducted in the “Measure” module within Consulface, with automatic detection of sagittal, transverse, and vertical planes. Available analyses included facial ratio, forehead–eye, nasal, chin–lip, orthodontic, and overall facial analyses, with adjustable anatomical landmarks. Reports were generated via “Export PDF Report,” containing patient information, scan date, selected analyses, and multi-view facial images (front, profile, and basal views).

In the Measurement Results section, we included the facial report, forehead–eye measurements, nasal measurements, chin–lip measurements, orthodontic measurements, and facial analysis. Each measurement has its own specific parameters.

In this study, we performed the following measurements: upper facial height, middle facial height, lower facial height, vertical facial proportion, total facial height, upper lip length, lower lip length, interpupillary distance, bizygomatic width, nasofrontal angle, nasolabial angle, mouth width, mentolabial angle, facial angle, distance from the labrale superius to the E-line, and distance from the labrale inferius to the E-line. The FacialPlan software was used to automatically calculate two indices describing vertical facial balance: Vertical Facial Proportion I and Vertical Facial Proportion II. Vertical Facial Proportion I was defined as the ratio of the upper facial third to the combined middle and lower thirds, whereas Vertical Facial Proportion II was defined as the ratio of the lower facial third to the combined upper and middle thirds. Both indices were expressed as dimensionless ratios, with higher values indicating a relative elongation of the corresponding facial third. The MetiSmile facial scanner (Shining 3D, Hangzhou, China) was calibrated prior to data collection according to the manufacturer’s standard procedure, using the supplied calibration board under controlled, uniform lighting conditions. This process ensured proper alignment and system accuracy before scanning. Calibration accuracy was verified by scanning a reference object with known dimensions to confirm the consistency and precision of the measurements [21].

**Figure 1:**
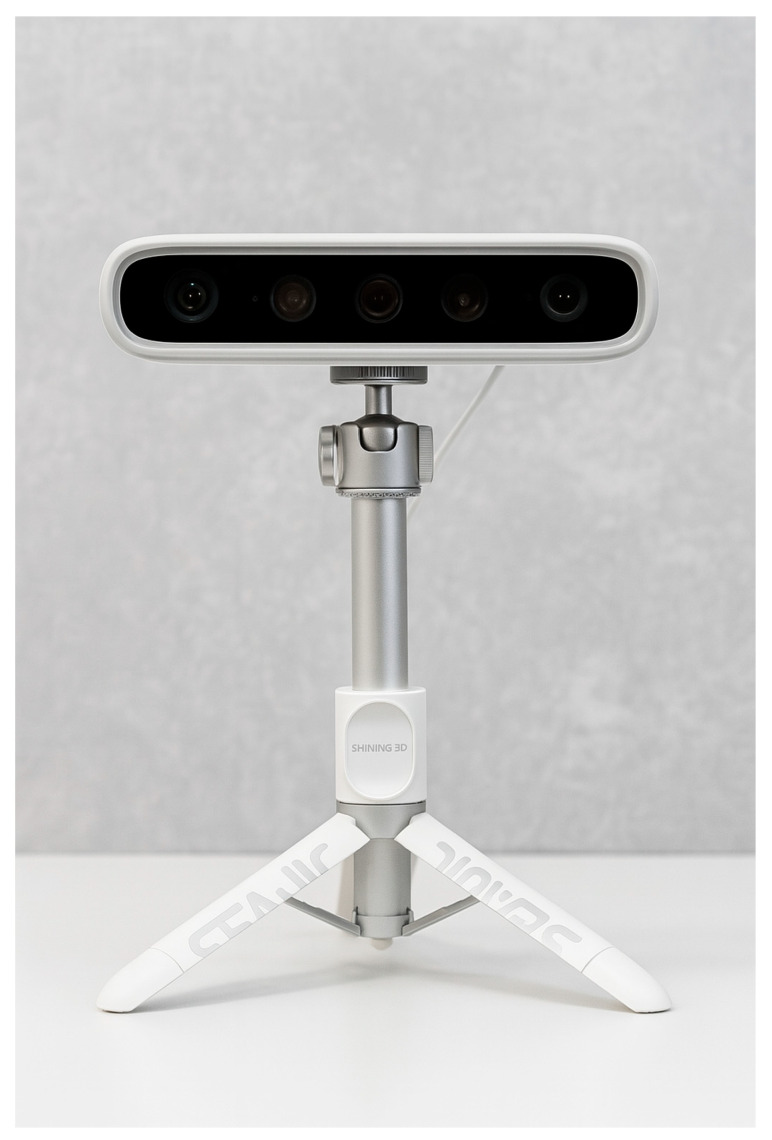
The MetiSmile Shining 3D Facial Scanner used in the facial anthropometric study—front view.

In Figure 1 below, we can see the MetiSmile Shining 3D Facial Scanner with which this study was conducted. The dual-camera system of the MetiSmile Shining 3D Facial Scanner is set on a sturdy support stand and includes an LED illumination source, a high-resolution texture camera, and an infrared depth sensor. The device uses the proprietary software package MetiSmile and is connected to a computer via USB.

**Figure 2:**
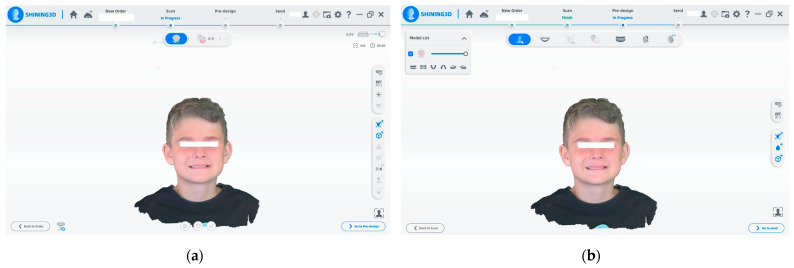
(**a**) Facial scanning in progress using Shining 3D and (**b**) completed facial scan and transition to pre-design. Note: Images are direct screenshots from the MetiSmile software (version V1.2.1.3), which does not allow high-resolution export.

In Figure 2, we describe and show the steps for scanning a patient. Enter the patient’s name to create a New Order and begin the facial scanning procedure. You can look up the patient’s profile if they have already had an intraoral or facial scan. Create a new profile for new patients. Click Scan to start the scanning procedure after the patient has been chosen. Once the scan is finished, click Send to upload the scan and continue to Pre-Design for evaluation. Click Send one more time to confirm. After the scan is finished, it will be added to the patient list for further access and use.

(a) The Shining 3D program is used for the initial facial scanning procedure. Throughout the scan, the 3D model is created in real time. The technology reconstructs the surface geometry of the face using a calibrated camera and structured light projection. The scanning process is displayed on the upper status bar, which tracks the progress.
(b) When the facial scan is complete, the pre-design stage begins. The software offers a precise 3D depiction of the patient’s face following data collection. For additional processing, simulation, or integration with intraoral and CBCT data, the user can proceed to the pre-design module.

**Figure 3:**
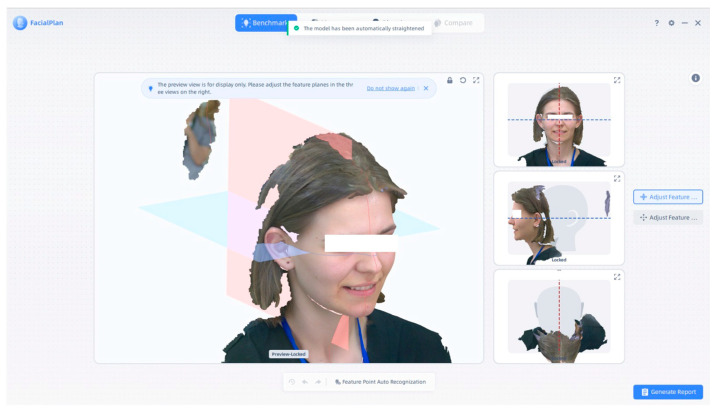
Processed 3D facial scan with aligned reference planes. Note: Images are direct screenshots from the MetiSmile software (version V1.2.1.3), which does not allow high-resolution export.

In Figure 3, we processed a subject’s three-dimensional facial scan using the MetiSmile Shining 3D Facial Scanner. The software interface shows facial feature recognition and automatic anatomical plane alignment.

**Figure 4:**
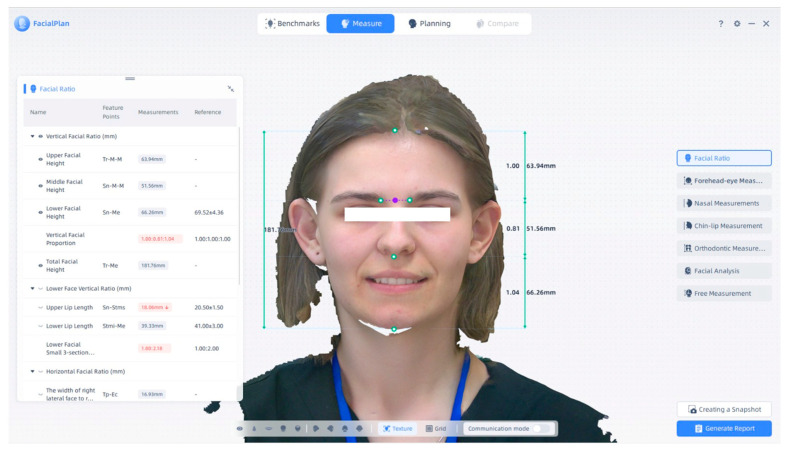
The MetiSmile Shining 3D Facial Scanner and FacialPlan software are used to analyze a scanned subject’s vertical and horizontal facial ratios. Note: Images are direct screenshots from the MetiSmile software, which does not allow high-resolution export.

There are several measurement options, as described above. Using the MetiSmile Shining 3D Facial Scanner and FacialPlan software, the vertical and horizontal facial proportions were analyzed. Figure 4 presents a screenshot from this program, showing the green vertical line used for measurements. Total facial height is 181.76 mm in this case. Upper facial height is 63.94 mm, marked by a green vertical line in the program; the middle facial height has a value of 51.56 mm, and the lower facial height has a value of 66.26 mm. The vertical facial proportions are 1.00:0.81:1.04. These measurements provide an in-depth anthropometric evaluation for facial morphology and orthodontic analysis.

**Figure 5:**
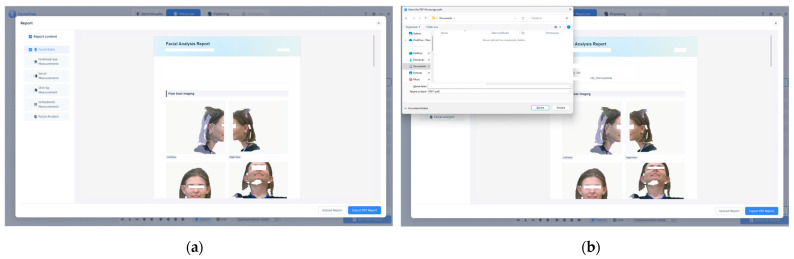
(**a**) Standardized facial scans, including left, right, frontal, and submental angles used for morphometric evaluation, are produced by the MetiSmile Shining 3D Facial Scanner and shown in the FacialPlan software interface. (**b**) An interface for exporting the 3D facial analysis report as a PDF file is also provided. Note: Images are direct screenshots from the MetiSmile software, which does not allow high-resolution export.

FacialPlan software (version V1.2.1.3) and the MetiSmile Shining 3D Scanner were used to automatically generate the facial analysis report. In Figure 5a, standardized 3D scans of the subject’s face from the frontal, submental, left, and right perspectives are included in the report, while Figure 5b shows the interface used to save and export the report as a PDF file. These illustrations facilitate the examination of facial symmetry, proportions, and structural harmony and are used as a guide for anthropometric assessment and orthodontic diagnosis.

**Figure 6:**
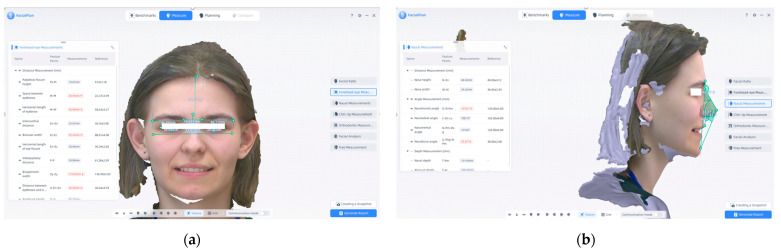
(**a**) forehead–eye measurements and (**b**) nasal measurements. Note: Images are direct screenshots from the MetiSmile software, which does not allow high-resolution export.

As shown in Figure 6a, forehead–eye measurements and (b) nasal measurements present an example of the measurements that can be performed using this program. (a) Forehead measurements were performed using the FacialPlan software based on 3D facial scans acquired with the MetiSmile Shining 3D Scanner. These measurements contribute to detailed facial morphometric evaluation, with applications in orthodontic diagnosis and aesthetic assessment.

**Figure 7:**
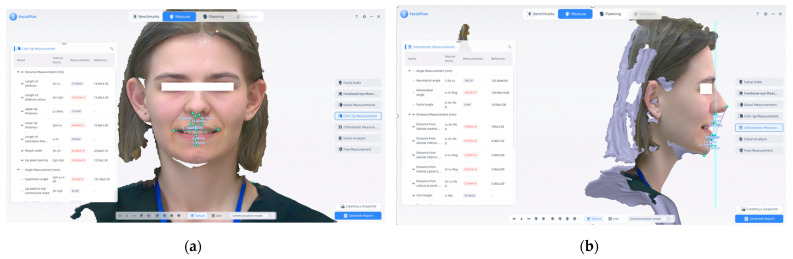
(**a**) Chin–lip measurements and (**b**) orthodontic measurements. Note: Images are direct screenshots from the MetiSmile software, which does not allow high-resolution export.

Figure 7 is another example of measurements that we can make in this program. (a) Chin–lip measurements were performed using the FacialPlan software on 3D facial scans acquired with the MetiSmile Shining 3D Scanner. These measurements are essential for assessing the vertical lip–chin proportion, soft tissue balance, and labial symmetry. (b) Orthodontic measurements were obtained from the profile view. These values provide critical data for evaluating facial convexity, lip protrusion, and skeletal profile, contributing to orthodontic and esthetic treatment planning.

**Figure 8:**
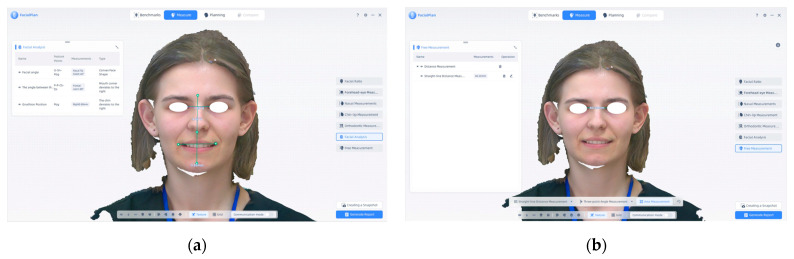
(**a**) Facial analysis and (**b**) free measurement. Note: Images are direct screenshots from the MetiSmile software, which does not allow high-resolution export.

In Figure 8, we have another type of measurement that we can make in this program, such as (a) facial analysis performed using the FacialPlan software, based on 3D scans acquired with the MetiSmile Shining 3D Scanner; (b) an example of a free-form linear measurement using the “Free Measurement” function in FacialPlan. One can freely measure any parameter desired by the doctor; for example, in the following image, we have drawn the interpupillary distance. This tool allows custom distance evaluations beyond the software’s standard anthropometric modules, offering additional flexibility for clinical and research purposes.

Facial Analysis Report is also included, in which we can observe deviations in facial symmetry, such as a convex facial profile, mouth corner asymmetry, and slight chin deviation to the right.

Upper facial height, middle facial height, lower facial height: Upper third: The height of the forehead (from trichion to glabella). Middle third—Height of the midface (glabella to subnasale). Lower third—Height of lower face (subnasale-gnathion). Upper facial height (UFH) (mm)—mean ± S.D.—62.12 ± 8.076. Middle facial height (MFH) (mm)—Mean ± S.D.—60.27 ± 4.867. Lower facial height (LFH) (mm)—mean ± S.D.—59.463 ± 5.3451 [22]. The normal values, taken directly from the MetiSmile Shining 3D Facial Scanner software, were 69.52 ± 4.36 mm for Lower Facial Height.

Vertical facial proportion is split equally between the middle, lower, and upper thirds. The center third runs from the glabella to the subnasale, the lower third from the subnasale to the soft tissue menton, and the upper third runs from the hairline to the glabella [23]. The normal value, taken directly from the MetiSmile Shining 3D Facial Scanner software, is 1.00:1.00:1.00.

Total Facial Height (TFH)—vertical separation between the chin (or the lowest point of the chin) and the hairline (or a line joining the topmost portion of the forehead). The formula for total facial height is TFH = hairline to chin distance. It can be further divided into more manageable parts, such as upper facial height (from the eyes to the hairline) and lower height of the face (from the eyes to the chin) [24].

Upper lip length—measured from the subnasal to the middle of the upper lip’s most inferior point [25]. Females typically have upper lip lengths between 18.0 and 22.0 mm, whereas males typically have upper lip lengths between 20.0 and 24.0 mm, according to Fonseca. According to a study by Hega et al., women’s upper lip length was 3.1 mm less than men’s [25]. Upper lip length at rest and when smiling—subnasale to stomion superius [26]. The normal value, taken directly from the MetiSmile Shining 3D Facial Scanner software, is 20.50 ± 1.50.

Lower lip length—at rest and when smiling—from stomion inferius to menton [26]. The normal value, taken directly from the MetiSmile Shining 3D Facial Scanner software, is 41.00 ± 3.00.

Interpupillary distance (IPD)—The facial measurement (in millimeters) in the horizontal plane between the geometric centers of the pupillary apertures, when the eyes are focused at optical infinity, is known as the far interpupillary distance (FIPD)**.** In the approximate spectacle (frontal) plane, this measurement is usually taken across the upper bridge of the nose. Typical FIPD values for adult (predominantly Caucasian/European) subjects are as follows, summarized from Pointer (1999): females = 60–61 mm (range 57–65 mm) and males = 63–64 mm (range 58–72 mm) [27]. The measurement of the distance between the centers of the eye’s pupils, i.e., between the centers of the irises, is known as interpupillary distance. The normal value, taken directly from the MetiSmile Shining 3D Facial Scanner software, is 61.28 ± 2.99.

Bizygomatic width—Measured from zygion to zygion, the mean bizygomatic width in the sample studied by Caton and Dixson was 142.62 ± 6.22 mm for men and 133.77 ± 5.56 mm for women. These values closely align with U.S. population averages reported in the same study, where the average male bizygomatic width was 140.67 ± 6.02 mm and the average female width was 131.81 ± 5.09 [28]. The normal value, taken directly from the MetiSmile Shining 3D Facial Scanner software, is 136.90 ± 5.02.

Nasofrontal angle—A vital component of nasal and facial profile aesthetics is the nasofrontal angle (NFA), which is created at the nasion by the junction of the nasal dorsum line and the glabella–nasion line. The optimal NFA, according to a quantitative analysis by Naini et al. (2016), is around 130°, with a wider range of 127° to 142° that is aesthetically acceptable [29]. The normal value, taken directly from the MetiSmile Shining 3D Facial Scanner software, is 130.00 ± 5.00.

Nasolabial angle—The angle formed by the tangent to the columella and the tangent to the upper lip. Normal values: 102°. Interpretation: high values => labial retrusion; low values => labial protrusion [30]. The normal value, taken directly from the MetiSmile Shining 3D Facial Scanner software, is 102.00 ± 8.00.

Mouth width—When the lips are relaxed and closed, the linear distance between the mouth’s outer corners (cheilions) is measured. Ubulu et al. (2020) reported that males had a significantly greater mean mouth width (58.91 ± 7.26 mm) than females (47.42 ± 6.10 mm) [31]. The normal value, taken directly from the MetiSmile Shining 3D Facial Scanner software, is 47.60 ± 3.70.

Mentolabial angle—The angle created at the sublabiale point by the intersection of two lines, one drawn from the sublabiale to the soft tissue pogonion (chin) and the other from the lower lip (labrale inferius), is sometimes referred to as the labiomental angle. The most aesthetically pleasant mentolabial angles, according to a quantitative study by Naini et al. (2017) [32] assessing male Caucasian profile shapes, fell between 107° and 118°. Angles below 98° or beyond 162° were deemed extremely unpleasant, while a wider acceptable range included up to 140° [32]. The normal value, taken directly from the MetiSmile Shining 3D Facial Scanner software, is 120.00 ± 10.00.

Facial angle is the angle on a lateral cephalogram between the Frankfort Horizontal plane (FH) and the line that connects Nasion (N) to Pogonion (Pog). The facial angle in boys and girls in the Obaidi and Abdul-Qadir (2007) study ranged from roughly 86.5° to 88.1° and 87.1° to 89.2°, respectively, between the ages of 11 and 14, with a notable increase in females at age 13 [33]. The normal value, taken directly from the MetiSmile Shining 3D Facial Scanner software, is 10.00 ± 5.00.

Distance from the labrale superius to the E-line: the normal value, taken directly from the MetiSmile Shining 3D Facial Scanner software, is 1.50 ± 0.50. Distance from the labrale inferius to the E-line: the normal value, taken directly from the MetiSmile Shining 3D Facial Scanner software, is 0.00 ± 0.00. The distances from the labrale superius and labrale inferius to the E-line were defined by Hellak et al. (2015) as the linear measurements taken perpendicularly from the most anterior points of the upper and lower lips, respectively, to Ricketts’ esthetic line that connects the pronasale and soft-tissue pogonion [34].

### 2.2. Statistical Analysis

Data entry was performed using Microsoft Excel, and statistical analyses were conducted in RStudio (version 4.3.1). The primary objective of the analysis was to explore correlations among soft-tissue facial anthropometric parameters and evaluate differences based on demographic variables such as age and sex.

All continuous variables were first tested for normality using the Shapiro–Wilk test. Based on the distribution, parametric or non-parametric methods were selected accordingly. Normally distributed variables were compared using Independent Samples *t*-tests, while non-normally distributed variables were assessed using Mann–Whitney *U* tests. For multiple group comparisons (across age strata), one-way ANOVA was applied. When homogeneity of variances was violated (as assessed by Levene’s test), Welch’s ANOVA was used. Significant ANOVA results were followed by post hoc pairwise comparisons using Tukey’s HSD test to control for Type I error. Spearman’s rank correlation coefficient (ρ) was used to evaluate relationships among facial dimensions, including vertical heights, transverse widths, lip parameters, and angular measurements. Correlations were interpreted with 95% confidence intervals, and significance was determined at *p* < 0.05. A priori power analysis based on a medium effect size (Cohen’s *d* ≈ 0.60), derived from intergroup differences in similar craniofacial studies, indicated that a minimum of 95 subjects would be required (α = 0.05, power = 0.80). The final sample of 90 participants was therefore considered near-optimal for detecting medium-sized effects in this cross-sectional analysis.

To assess intraobserver reliability, 20% of the scans were randomly re-analyzed after a four-week interval by the same examiner. Intraclass correlation coefficients (ICC) were computed for key measurements. ICC values above 0.75 were considered to reflect acceptable reproducibility.

All statistical tests were two-tailed with a significance threshold of *p* < 0.05. Where applicable, effect sizes (Cohen’s *d*, correlation coefficients) were reported to supplement *p*-values and enhance interpretation of clinical relevance.

Sex-based comparisons were deemed experimental because of the age disparity between the groups.

## 3. Results

The cohort comprised 90 individuals, including 57 (63%) females and 33 (37%) males, with a median age of 14 years (10, 24). Regarding facial dimensions, the median upper facial height was 56.2 mm (53.1, 59.4), the middle facial height was 55.7 mm (51.9, 60.1), and the lower facial height was 60 mm (56, 64). All participants (90 [100%]) exhibited a vertical facial proportion classified as level 1.

The median vertical facial proportion ratios were 1.01 (0.92–1.06) for the upper region (Vertical Facial Proportion I) and 1.08 (0.99–1.15) for the lower region (Vertical Facial Proportion II). These indices were automatically calculated by the FacialPlan software. Vertical Facial Proportion I represents the ratio of the upper facial third to the combined middle and lower thirds (trichion–glabella, glabella–subnasale, subnasale–soft-tissue menton), whereas Vertical Facial Proportion II represents the ratio of the lower facial third to the combined upper and middle thirds. Both indices are dimensionless, with higher values indicating a relative elongation of the corresponding facial third.

The median total facial height was 173 mm (162, 180). The median upper lip length was 15.88 mm (13.77, 18.06), while the median lower lip length was 37.1 mm (33.2, 41.2). Interpupillary distance showed a median of 58.8 mm (55.4, 61.7), and bizygomatic width had a median of 110 mm (104, 115).

Facial angles included a median nasofrontal angle of 144° (140, 148) and a median nasolabial angle of 116° (108, 123). The median mouth width was 51 mm (47, 56), and the median mentolabial angle was 137° (126, 149). The facial angle was considerably smaller, with a median of 14.2° (10.0, 19.7). Additionally, median distances from Labrale superius and Labrale inferius to the E-line were 3.8 mm (1.6, 6.6) and 2.57 mm (1.29, 4.54), respectively. (Table 1).

In this cohort of ninety subjects (n = 90), Spearman’s rank correlations revealed distinct patterns among vertical facial dimensions, lip-perioral measures, and angular variables.

Among the vertical linear measures, age demonstrated moderate to strong positive associations with most absolute dimensions. Age correlated most strongly with total facial height (r = 0.690, 95% CI 0.563–0.785, *p* < 0.001), middle facial height (r = 0.631, 95% CI 0.488–0.741, *p* < 0.001), and lower facial height (r = 0.615, 95% CI 0.467–0.729, *p* < 0.001), and to a lesser extent with upper facial height (r = 0.334, 95% CI 0.136–0.506, *p* = 0.001). A weaker but still significant relationship was seen between age and the lower-third proportion index (vertical facial proportion II: r = 0.258, 95% CI 0.054–0.442, *p* = 0.014), whereas age was not significantly associated with the upper-third proportion index (vertical facial proportion I: r = 0.198, 95% CI −0.009–0.389, *p* = 0.061). (Table 2).

Turning to lip morphology and transverse measures, upper and lower lip lengths were moderately intercorrelated (r = 0.528, 95% CI 0.360–0.663, *p* < 0.001). Upper lip length also correlated with interpupillary distance (r = 0.445, 95% CI 0.262–0.597, *p* < 0.001) and bizygomatic width (r = 0.293, 95% CI 0.091–0.471, *p* = 0.005), but not with mouth width (r = −0.052, 95% CI −0.256–0.157, *p* = 0.625). Lower lip length showed similar associations—strongly with interpupillary distance (r = 0.527, 95% CI 0.359–0.662, *p* < 0.001) and with bizygomatic width (r = 0.584, 95% CI 0.429–0.706, *p* < 0.001), and modestly with mouth width (r = 0.278, 95% CI 0.075–0.459, *p* = 0.008). The horizontal distance between Labrale superius and the E-line was significantly associated with mouth width (r = 0.665, 95% CI 0.531–0.767, *p* < 0.001) as well as with lower lip length (r = 0.241, 95% CI 0.036–0.427, *p* = 0.022), whereas its counterpart at Labrale inferius correlated strongly with mouth width (r = 0.490, 95% CI 0.315–0.633, *p* < 0.001) but not significantly with lower lip length (r = 0.126, 95% CI −0.083–0.325, *p* = 0.235). (Table 3).

The two vertical proportion indices themselves exhibited opposing relationships with the linear heights. The upper-third index (proportion I) was strongly and inversely correlated with upper facial height (r = −0.611, 95% CI −0.726–0.462, *p* < 0.001) and positively with middle facial height (r = 0.539, 95% CI 0.374–0.671, *p* < 0.001), but showed no significant association with lower facial height (r = 0.090, 95% CI −0.119–0.292, *p* = 0.398) or with total facial height (r = 0.025, 95% CI −0.183–0.231, *p* = 0.812). The lower-third index (proportion l) was moderately negatively related to upper facial height (r = −0.438, 95% CI −0.591–0.254, *p* < 0.001) and positively to lower facial height (r = 0.600, 95% CI 0.449–0.718, *p* < 0.001), with smaller but significant correlations to middle facial height (r = 0.247, 95% CI 0.042–0.432, *p* = 0.019) and total facial height (r = 0.218, 95% CI 0.011–0.407, *p* = 0.039). (Table 4).

Angular measures of nasal and lower-face form demonstrated limited interdependence. The nasofrontal angle showed no significant correlations with either the nasolabial (r = −0.001, 95% CI −0.208–0.206, *p* = 0.990) or mentolabial angles (r = 0.023, 95% CI −0.185–0.229, *p* = 0.827). In contrast, the nasolabial and mentolabial angles were moderately positively related (r = 0.360, 95% CI 0.165–0.528, *p* < 0.001), and the facial angle correlated positively with the nasolabial angle (r = 0.385, 95% CI 0.193–0.548, *p* < 0.001) but inversely with the mentolabial angle (r = −0.391, 95% CI −0.553–0.200, *p* < 0.001).

In this cohort of 90 individuals, comprising 57 (63.3%) females and 33 (36.7%) males, several facial morphological differences emerged between sexes. Females (median age 22.0 years, IQR 12.0–27.0) were significantly older than males (median age 11.0 years, IQR 9.0–16.0; *p* = 0.002).

Regarding vertical facial dimensions, females exhibited significantly greater median middle facial height (57.1 mm, IQR 53.2–60.8) compared to males (53.5 mm, IQR 49.0–58.0; *p* = 0.002), as well as greater total facial height (175.2 mm, IQR 167.6–180.1 versus 165.4 mm, IQR 152.9–179.4; *p* = 0.045). Upper facial height (females 57.2 mm, IQR 54.5–59.4; males 55.4 mm, IQR 47.9–59.0; *p* = 0.105) and lower facial height (females 60.3 mm, IQR 56.3–63.5; males 59.2 mm, IQR 53.7–65.5; *p* = 0.940) did not differ significantly between sexes. Additionally, vertical facial proportion indices showed no statistically significant sex-based differences, although the lower facial proportion (vertical facial proportion II) trended higher among males (1.1, IQR 1.0–1.2 versus 1.0, IQR 1.0–1.1 in females; *p* = 0.067).

Lip dimensions and transverse facial widths revealed no significant sexual dimorphism. Specifically, upper lip length (females 15.5 mm, IQR 13.7–17.6; males 16.2 mm, IQR 14.6–18.6; *p* = 0.175), lower lip length (females 38.0 mm, IQR 35.1–41.1; males 36.6 mm, IQR 32.1–42.8; *p* = 0.368), interpupillary distance (females 59.1 mm, IQR 56.1–62.1; males 58.6 mm, IQR 55.0–61.7; *p* = 0.506), and bizygomatic width (females 110.3 mm, IQR 105.2–115.5; males 107.8 mm, IQR 101.9–112.9; *p* = 0.187) were comparable across gender groups. Mouth width similarly showed no significant difference between females (52.0 mm, IQR 47.4–56.3) and males (49.7 mm, IQR 43.9–54.1; *p* = 0.152).

Angular measurements indicated subtle yet significant sexual differences. Females demonstrated a significantly greater nasofrontal angle (144.6°, IQR 141.2–150.0) compared to males (142.1°, IQR 138.6–146.4; *p* = 0.025). Conversely, males exhibited a significantly larger nasolabial angle (122.1°, IQR 115.2–125.2 versus 114.0°, IQR 107.0–120.2 in females; *p* = 0.001). Other angular parameters, including mentolabial angle (females 136.6°, IQR 124.7–145.4; males 139.9°, IQR 130.5–150.0; *p* = 0.171) and overall facial angle (females 13.7°, IQR 9.5–18.5; males 15.0°, IQR 10.1–20.8; *p* = 0.288), revealed no statistically significant differences between sexes.

Finally, the position of lips relative to the E-line differed only for the upper lip, with females showing significantly greater median distances from Labrale superius to the E-line (4.5 mm, IQR 2.1–7.1) than males (2.8 mm, IQR 1.1–4.9; *p* = 0.041). The analogous lower lip measurement showed no significant sex difference (females 2.6 mm, IQR 1.4–4.8; males 2.5 mm, IQR 1.3–3.6; *p* = 0.269). Considering the age disparity (females older than males), these intersex differences should be regarded with caution, as they may partially represent age-related maturity. (Table 5).

**Figure 9:**
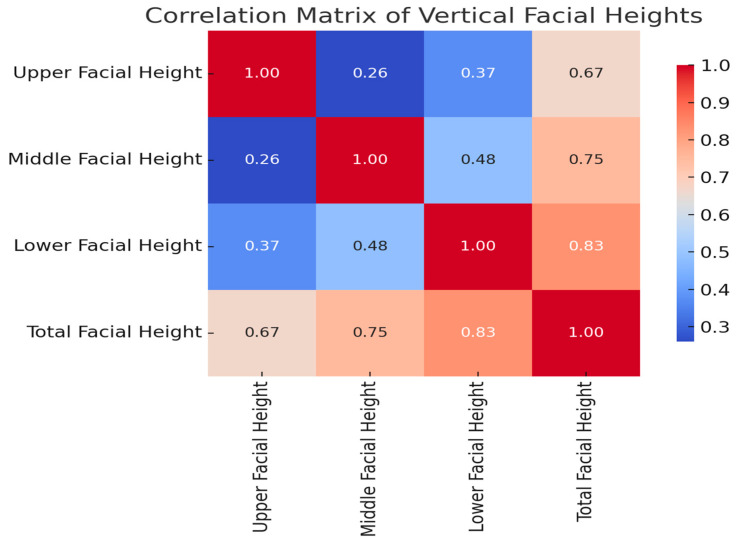
Correlation matrix of vertical facial heights.

Figure 9 presents a heatmap illustrating Spearman’s correlation coefficients among key vertical facial dimensions: upper facial height, middle facial height, lower facial height, and total facial height. The color gradient reflects the strength of correlation, with red tones indicating stronger positive relationships. As shown, total facial height was strongly correlated with both lower facial height (r = 0.83) and middle facial height (r = 0.75), underscoring their dominant contribution to overall vertical facial development. Upper facial height exhibited a moderate correlation with total facial height (r = 0.67) and weaker correlations with the middle (r = 0.26) and lower facial heights (r = 0.37), supporting the observation that upper facial structures stabilize earlier in growth compared to the lower facial third. Clinical relevance: These patterns confirm the role of middle and lower facial dimensions as primary drivers of vertical facial growth during adolescence and highlight the importance of tracking these parameters in orthodontic diagnostics and timing of interventions.

## 4. Three-Dimensional Facial Visualization and Measurement Reports

Using the MetiSmile Shining 3D Scanner and the FacialPlan software, 3D facial scans were acquired and automatically analyzed to generate standardized measurement reports. The software provided both vertical and horizontal linear parameters, as well as angular and proportional indices relevant to orthodontic and anthropometric assessment.

Figure 4 illustrates an example of the vertical facial height segmentation as displayed in the software interface. The total facial height measured in this representative case was 181.76 mm, with the upper, middle, and lower facial heights measuring 63.94 mm, 51.56 mm, and 66.26 mm, respectively. The vertical facial proportion for this example was therefore 1.00:0.81:1.04. These measurements demonstrate the approach used for detailed anthropometric evaluation of facial morphology and provide the basis for quantitative orthodontic assessment.

Figure 5 presents examples of the automatically generated facial analysis report in the MetiSmile system. Panel (a) includes standardized 3D facial views from the frontal, submental, left, and right perspectives, while panel (b) shows the interface used to export the report as a PDF file. These visualizations facilitate the evaluation of facial symmetry, proportions, and overall structural harmony, serving as a reference for anthropometric assessment and orthodontic diagnosis.

Figure 6 shows examples of forehead–eye and nasal measurements performed using the FacialPlan software based on 3D facial scans acquired with the MetiSmile Shining 3D Scanner. Panel (a) shows forehead and eye measurements, while panel (b) depicts nasal morphometric parameters automatically generated by the system. These measurements provide a detailed morphometric characterization of the upper facial region and are valuable for both orthodontic diagnosis and aesthetic evaluation.

Figure 7 presents additional examples of measurements that can be performed using the FacialPlan software. Panel (a) illustrates chin–lip measurements used to assess vertical lip–chin proportions, soft-tissue balance, and labial symmetry. Panel (b) displays orthodontic measurements obtained from the profile view, which provide essential data for evaluating facial convexity, lip protrusion, and the overall skeletal profile. These parameters contribute to comprehensive orthodontic and aesthetic treatment planning.

Figure 8 illustrates additional types of measurements available in the FacialPlan software. Panel (a) shows an example of automated facial analysis, while panel (b) demonstrates the “Free Measurement” function, which enables custom linear measurements beyond the predefined anthropometric modules. In the example shown, the interpupillary distance was measured manually to illustrate the flexibility of this feature. Such tools allow clinicians and researchers to perform customized evaluations tailored to specific diagnostic or investigative objectives.

Together, Figure 4, Figure 5, Figure 6, Figure 7 and Figure 8 illustrate representative examples of the facial landmarks and measurement parameters analyzed in this study, providing a comprehensive overview of the digital anthropometric workflow used for soft-tissue evaluation.

In summary, the results presented above provide a detailed overview of the three-dimensional soft-tissue measurements and digital facial analysis workflow used in this study. These findings form the basis for the following discussion on their clinical relevance, limitations, and implications for orthodontic diagnosis.

## 5. Discussion

This study examined the relationship between demographic factors and craniofacial anthropometric measurements in a Romanian orthodontic population using structured-light 3D facial scanning technology. The high-resolution, non-invasive assessments provided by the MetiSmile Shining 3D scanner enabled precise evaluation of soft tissue landmarks, offering clinically meaningful insights into facial morphology [5,8].

Since CBCT is regarded as the gold standard, it is imperative to continuously evaluate the patient’s exposure to ionizing radiation and any resulting biological harm. This is why radiation-free diagnostic methods like MRIs (magnetic resonance imaging) and ultrasounds are being developed and improved [35].

When traditional 2D imaging methods are insufficient, CBCT should only be utilized after careful assessment, particularly with regard to patient exposure [36].

Rapid advancements in medical imaging are causing a revolution in the field of medicine. Digital dentistry is evolving at a rapid pace, as seen by the development of cone-beam computed tomography (CBCT), intraoral and facial scanners, dental 3D printing, and artificial intelligence. Differentiating or segmenting objects, organs, or structures from their surrounding background is often required in medical picture studies [37].

Typical diagnostic limitations of conventional dental panoramic radiography and plain film radiography include expansion and distortion, setting mistakes, positional artifacts, and the lack of information about the size of the bone in the buccolingual direction [38]. As dental imaging technology continues to advance, orthodontics is evolving from diagnosis to treatment planning [39].

Due to the significance of this most anterior part of the aerodigestive tract, which plays vital roles in speech, mastication, taste, deglutition, aesthetics, and general well-being, dentomaxillary pathology is a significant medical concern worldwide. The integrity of the oral cavity will be impacted by pathological abnormalities in the teeth and jawbones, which will have major ramifications for all these processes. Any local illness has the potential to produce general and regional issues that affect the entire body and have a significant influence on population health [40].

In actuality, since combined data can achieve an 85% diagnosis accuracy, the doctor and the radiologist must collaborate as a team when staging oral cavity pathogenic entities [41].

One of the essential components of the body for self-recognition is the face. It is the primary individual factor that determines an individual’s physical appearance. Every human face is different and adds to a person’s identity. Numerous studies have examined the significance of physical and facial attractiveness and its relationship to decisions about hiring, first impressions, peer pressure, voting and juror selection, and social interactions, including dating decisions [22].

A study by Rathi and Chhetri (2018) [22] evaluated the upper, middle, and lower face height measurements of males and females in a Biratnagar teaching hospital. Their results showed that, in comparison to females, males had higher mean values for each of the three dimensions of face height. In particular, the average upper face height (UFH) for males was 64.00 ± 8.705 mm, whereas the average for females was 61.20 ± 7.613 mm. Additionally, males had a higher middle facial height (MFH) (60.77 ± 4.854 mm) than females (60.02 ± 4.872 mm). The lower facial height (LFH) of males and females was measured to be 60.598 ± 5.5727 mm and 58.903 ± 5.1589 mm, respectively. These findings demonstrate that there is sexual dimorphism in the measurements of face height, with males often exhibiting bigger facial proportions. These results highlight the importance of taking sex-specific anthropometric standards into account when assessing facial harmony and treatment planning, which is crucial for clinicians working in orthodontic, prosthodontic, and surgical planning.

There are significant differences in the human face based on age, sex, race, and ethnicity. Harmony, symmetry, soft-to-bony tissue ratios, and lower facial height ratios are some examples of the variations. Although the definition of facial beauty varies from person to person, it does have a quantifiable ratio known as the golden ratio. The golden proportion was first proposed for use in dentistry by Lombardi, although he also said, “It has proved too strong for dental use.” According to Snow, the idea of the golden percentage can be applied to the diagnosis and development of symmetry, dominance, and proportion for a grin that is aesthetically beautiful [22].

According to Vitruvius, who listed the facial and body proportions, a man’s height is ten times the distance between his hairline and the inferior side of his chin. He said that the face is made up of the following: 1/3 of the face is from the chin to just behind the nostrils. A third of the face is made up of the area directly under the nose and the eyebrows. The hairline and eyebrows make up about one-third of the face [42].

According to a study by Jain et al. [43] in India, the lower third of the face is larger than the middle third, with respective values of 55.37% and 44.63%. This is in good agreement with Powell and Humphries’ (1984) study of the North American population, which found that the lower and middle thirds of the face had values of 53% and 47%, respectively [43].

According to a study by Sadaccharan et al., women’s forehead heights are higher at 54.23 mm than men’s (51.95 mm) [44].

According to photographic research by Husein et al. on Indian American women’s facial height (54.2 mm), the midface height of these women (58.1 mm) displayed a lower value [45].

According to the literature that is now accessible, the middle and higher thirds are less than the lower third, and the middle third is frequently smaller than the top third among Caucasians. In East Asians, the upper third of the face is smaller than the lower third, while the middle third is frequently larger than the upper third and equal to the lower third [46].

The MetiSmile system (Shining 3D, Hangzhou, China) used in this study represents a state-of-the-art, structured-light, infrared-based 3D facial scanner designed specifically for orthodontic applications. Utilizing a VCSEL (vertical-cavity surface-emitting laser) projector in combination with three infrared cameras and a 5 MP HD texture camera, the device captures high-resolution facial geometry with an accuracy of approximately 50 μm in under 10 s [17,47]. As an extraoral scanner, MetiSmile offers a non-invasive alternative for acquiring soft tissue morphology, which can be seamlessly integrated with intraoral scans to enhance diagnostic and treatment workflows [17,48]. Furthermore, its AI-driven capabilities—including automatic lip/tooth segmentation, cephalometric analysis, and mandibular movement tracking—offer added value in orthodontic planning and facial aesthetic evaluation [17,49]. These technological advantages underscore the clinical potential of structured-light 3D scanning as a high-precision, radiation-free solution for modern digital orthodontics [48,49].

Several clinical implications emerge from the findings. Growth Monitoring: Strong correlations between age and key vertical facial dimensions (especially total, middle, and lower facial height) support the use of 3D scanning for longitudinal growth assessment. This is particularly valuable for evaluating treatment timing in growing patients. Esthetic Diagnosis: Differences in angular measurements (e.g., nasofrontal and nasolabial angles) and lip protrusion (distance from E-line) highlight how 3D analysis can assist in identifying soft tissue imbalances that may influence facial esthetics and orthodontic treatment planning. Sex-Based Norms: Subtle yet statistically significant differences between males and females in facial heights and angles underscore the importance of sex-specific reference data when planning interventions, especially in orthognathic surgery or esthetic orthodontics. Treatment Individualization: The vertical facial proportion indices (upper-third and lower-third ratios) provide a nuanced understanding of facial harmony beyond absolute values. These metrics may guide individualized treatment goals based on proportion rather than population norms alone. Radiation-Free Assessment: Since 3D facial scanning does not involve ionizing radiation, it can be safely repeated during treatment to monitor progress or post-treatment changes, making it highly suitable for use in pediatric and adolescent patients. Integration into Digital Workflow: The collected data can be incorporated into virtual treatment simulations, enabling interdisciplinary planning across orthodontics, prosthodontics, and maxillofacial surgery.

Finally, the use of structured-light 3D scanning demonstrated several clinical advantages: radiation-free imaging, high accuracy, reproducibility, and patient comfort. Devices like the MetiSmile scanner allow for comprehensive 3D facial data collection without the risks associated with ionizing radiation [5,8].

Consistent with our findings of age-driven increases in facial height, Adekunle et al. (2022) reported significantly higher upper, middle, and lower facial heights in a Nigerian adult sample (mean ULH 69.1 ± 5.9 mm, MFH 49.9 ± 3.6 mm, LFH 67.9 ± 6.1 mm) [50].

Wilson, Medapati, and Segwapa (2023) [51] reported that young Black South African women exhibited a relatively shorter upper facial third compared to the middle and lower thirds, which parallels our cohort’s consistent Level 1 vertical proportion classification. These findings underscore both proportional stability and potential ethnic variability in facial morphology [51]. Anatolia’s adolescent nasal anthropometry confirmed our sex-specific angular findings by revealing substantial sex differences in nasofrontal and nasolabial angles (*p* < 0.001) [52].

Significant sex differences were discovered in a complete photogrammetric analysis of adolescents: females exhibited larger nasal angles overall, greater mentolabial angles, and wider nasofrontal angles (140.7° ± 5° vs. boys 138.2° ± 7.8°; *p* < 0.05). This finding supports the nasal-angular sex differences in adolescence and is consistent with our result that females have bigger nasofrontal angles [53].

In a study of 50 Garhwali people between the ages of 18 and 21, the mentolabial angle was wider in males (*p* = 0.001 and *p* = 0.002, respectively), and the nasofrontal angle was wider in females (141.9° ± 6°). This finding supports our finding that females had wider nasofrontal angles and points to consistent sexual patterns across populations [54].

All facial angles, including the nasolabial, mentolabial, facial, and nasofrontal, were statistically greater in females among adults in central Romania (nasolabial: 105.3° vs. 102.2°, *p* = 0.00002; mentolabial: 126.1° vs. 118.3°, *p* = 0.0000097; facial: 170.3° vs. 168.8°, *p* = 0.0033 *), confirming that there are angular disparities based on sex, particularly that female angles are often greater [55].

This technology is especially useful in pediatric and adolescent populations, where longitudinal monitoring is essential. Overall, structured-light scanning provides an effective, non-invasive means to assess facial growth, symmetry, and esthetic balance in orthodontic care. In addition, recent studies have incorporated artificial intelligence-powered diagnostic platforms, such as WebCeph [56,57,58], which represent a paradigm shift in modern orthodontics by enabling precise, automated cephalometric analysis, complementing innovations like the facial scanning technology employed in the present research. Recent investigations have validated the precision and clinical viability of virtually created 3D-printed surgical splints for orthognathic repositioning, affirming the dependability of digital processes in craniofacial analysis [59].

## 6. Study Limitations and Future Perspectives

This study has a number of shortcomings in spite of its advantages. Sample Demographics: The cohort may not be representative of other ethnic groups or populations because it was selected from a single clinical site in Romania. Larger and more varied sample sizes should be used in future research. Cross-Sectional Design: Because the study only records a single moment in time, it is not possible to draw conclusions about the growth paths of specific individuals. Age-related morphological changes would be best captured by a longitudinal approach. Age Imbalance: Some sex-based comparisons may have been impacted by a notable age gap between male and female participants, particularly in dimensions that are sensitive to pubertal maturation. Measuring Software Variability: Despite the great accuracy of the MetiSmile software, little variation in landmark identification and operator modifications could result in very little variability. Clinical Diversity and Exclusion Criteria: Results are not applicable to orthodontic patients with syndromic or post-surgical facial abnormalities since subjects with craniofacial anomalies were not included. To improve the development of comprehensive virtual patients, future studies should integrate intraoral and CBCT datasets, investigate ethnic-specific norms, and include longitudinal data. Further analysis of dynamic facial expressions with this technique may also yield additional information about morphometrics that is useful to functional analysis. This study used a cross-sectional methodology, allowing for the identification of relationships but precluding the establishment of causal conclusions or developmental trajectories. Future longitudinal investigations are necessary to confirm age-related morphological changes and to monitor individual face development patterns over time. The age disparity between genders may serve as a residual confounding variable in sex-based comparisons. Subsequent research should include age-matched cohorts or use age-adjusted statistical techniques to mitigate this impact. The wide age range of participants (9–27 years) encompasses many developmental phases, potentially affecting craniofacial measures. Future research should include stratified analyses or longitudinal designs to more accurately reflect stage-specific development patterns. A further drawback is the absence of a designated control group (e.g., a non-orthodontic population), which constrains the capacity to differentiate treatment-related effects from population-specific morphological traits. Subsequent studies need to include matched control groups or multicenter cohorts to improve external validity and comparability. The research used a convenience sample of orthodontic patients from one clinical location, potentially introducing selection bias and limiting population representativeness. Subsequent research should use randomized or multicenter samples to enhance external validity. All scans were conducted with a neutral facial expression to maintain consistency; nevertheless, this static evaluation does not represent dynamic soft-tissue motions during smiling or functional activities. Future investigations should use dynamic or expression-based three-dimensional analysis to improve the therapeutic relevance of face morphometry. The current investigation was confined to soft-tissue morphology without direct skeletal validation (CBCT or cephalometric correlation), hence limiting comprehensive anatomical interpretation. Future research should use multimodal datasets that merge 3D face scans with skeletal imaging to improve the comprehension of soft-tissue–bone interactions. The exclusion of individuals with craniofacial defects or a history of face surgery limits the direct generalizability of the results to syndromic, post-surgical, or otherwise complicated orthodontic patients. Future research must include a broader range of patient demographics to improve clinical usefulness and generalizability. Certain correlation coefficients had broad confidence intervals, indicating diminished accuracy and implying that these correlations need cautious interpretation. Augmenting the sample size in subsequent research may facilitate the attainment of smaller confidence intervals and more reliable results. This research did not conduct independent inter-device accuracy validation; scanner precision relied on previously published assessments and manufacturer standards. Subsequent research should include empirical cross-device comparisons to validate measurement accuracy and consistency.

## 7. Conclusions

This study validates the clinical applicability of structured-light 3D facial scanning for evaluating craniofacial anthropometric parameters in orthodontics. Strong correlations between age and key vertical facial dimensions—particularly total, middle, and lower facial height—support its use for longitudinal growth monitoring and optimal treatment timing in growing patients. Differences in angular measurements (e.g., nasofrontal and nasolabial angles) and lip protrusion from the E-line highlight the role of 3D analysis in detecting soft tissue imbalances relevant to esthetic diagnosis and treatment planning. Subtle yet statistically significant sex-based differences in certain facial heights and angles underscore the importance of sex-specific reference data, especially for orthognathic surgery and esthetic orthodontics. Vertical facial proportion indices (upper- and lower-third ratios) provide a more nuanced assessment of facial harmony, enabling individualized treatment objectives beyond population norms. As a radiation-free, repeatable method, 3D scanning is particularly suitable for pediatric and adolescent patients and can be integrated into digital workflows for interdisciplinary planning in orthodontics, prosthodontics, and maxillofacial surgery. Overall, 3D facial morphometry offers a non-invasive, precise, and esthetically oriented tool that enhances understanding of soft tissue relationships and facial proportions, facilitating customized, patient-centered orthodontic care.

## Figures and Tables

**Table 1:** Median values and interquartile ranges for vertical, transverse, lip, and angular facial parameters (in millimeters [mm] or degrees [°]) in 3D-scanned subjects.

Characteristic	N = 90
Gender	
F	57 (63%)
M	33 (37%)
Age	14 (10, 24)
Upper Facial Height	56.2 (53.1, 59.4)
Middle Facial Height	55.7 (51.9, 60.1)
Lower Facial Height	60 (56, 64)
Vertical Facial Proportion I-upper region	1.01 (0.92, 1.06)
Vertical Facial Proportion II-lower region	1.08 (0.99, 1.15)
Total Facial Height	173 (162, 180)
Upper Lip Length	15.88 (13.77, 18.06)
Lower Lip Length	37.1 (33.2, 41.2)
Interpupillary distance	58.8 (55.4, 61.7)
Bizygomatic width	110 (104, 115)
Nasofrontal angle	144 (140, 148)
Nasolabial angle	116 (108, 123)
Mouth width	51 (47, 56)
Mentolabial angle	137 (126, 149)
Facial angle	14.2 (10.0, 19.7)
Distance from Labrale superius to E-line	3.8 (1.6, 6.6)
Distance from Labrale inferius to E-line	2.57 (1.29, 4.54)

Note: Values are presented as n (%) for categorical variables and as median (Q1, Q3) for continuous variables. Linear dimensions are expressed in millimeters (mm), angular measurements in degrees (°), and age in years. Vertical Facial Proportion I and Vertical Facial Proportion II correspond to automated proportional indices generated by the FacialPlan software, representing the relative balance of the upper and lower facial thirds, respectively. Values are unitless ratios derived from vertical facial height segments.

**Table 2:** Correlations among age and vertical facial dimensions.

	Age	Upper Facial Height	Middle Facial Height	Lower Facial Height	Vertical Facial Proportion I	Vertical Facial Proportion II	Total Facial Height
Age	—						
Upper Facial Height	r = 0.334 (95% CI: 0.136 to 0.506), *p* = 0.001	—					
Middle Facial Height	r = 0.631 (95% CI: 0.488 to 0.741), *p* < 0.001	r = 0.257 (95% CI: 0.053 to 0.441), *p* = 0.014	—				
Lower Facial Height	r = 0.615 (95% CI: 0.467 to 0.729), *p* < 0.001	r = 0.367 (95% CI: 0.173 to 0.534), *p* < 0.001	r = 0.479 (95% CI: 0.302 to 0.624), *p* < 0.001	—			
Vertical Facial Proportion I	r = 0.198 (95% CI: −0.009 to 0.389), *p* = 0.061	r = −0.611 (95% CI: −0.726 to −0.462), *p* < 0.001	r = 0.539 (95% CI: 0.374 to 0.671), *p* < 0.001	r = 0.090 (95% CI: −0.119 to 0.292), *p* = 0.398	—		
Vertical Facial Proportion II	r = 0.258 (95% CI: 0.054 to 0.442), *p* = 0.014	r = −0.438 (95% CI: −0.591 to −0.254), *p* < 0.001	r = 0.247 (95% CI: 0.042 to 0.432), *p* = 0.019	r = 0.600 (95% CI: 0.449 to 0.718), *p* < 0.001	r = 0.622 (95% CI: 0.476 to 0.734), *p* < 0.001	—	
Total Facial Height	r = 0.690 (95% CI: 0.563 to 0.785), *p* < 0.001	r = 0.667 (95% CI: 0.534 to 0.768), *p* < 0.001	r = 0.748 (95% CI: 0.640 to 0.827), *p* < 0.001	r = 0.833 (95% CI: 0.756 to 0.887), *p* < 0.001	r = 0.025 (95% CI: −0.183 to 0.231), *p* = 0.812	r = 0.218 (95% CI: 0.011 to 0.407), *p* = 0.039	—

Note: “Vertical Facial Proportion I” and “Vertical Facial Proportion II” represent two distinct proportional indices calculated from different facial height segment ratios.

**Table 3:** Correlations among lip dimensions, facial widths, and labrale E-line distances.

	Upper Lip Length	Lower Lip Length	Interpupillary Distance	Bizygomatic Width	Mouth Width	Distance From Labrale Superius to E-Line	Distance From Labrale Inferius to E-Line
Upper lip length	—						
Lower lip length	r = 0.528 (95% CI: 0.360 to 0.663), *p* < 0.001	—					
Interpupillary distance	r = 0.445 (95% CI: 0.262 to 0.597), *p* < 0.001	r = 0.527 (95% CI: 0.359 to 0.662), *p* < 0.001	—				
Bizygomatic width	r = 0.293 (95% CI: 0.091 to 0.471), *p* = 0.005	r = 0.584 (95% CI: 0.429 to 0.706), *p* < 0.001	r = 0.845 (95% CI: 0.773 to 0.895), *p* < 0.001	—			
Mouth width	r = −0.052 (95% CI: −0.256 to 0.157), *p* = 0.625	r = 0.278 (95% CI: 0.075 to 0.459), *p* = 0.008	r = 0.487 (95% CI: 0.311 to 0.631), *p* < 0.001	r = 0.618 (95% CI: 0.471 to 0.731), *p* < 0.001	—		
Distance from Labrale superius to E-line	r = −0.201 (95% CI: −0.392 to 0.006), *p* = 0.057	r = 0.241 (95% CI: 0.036 to 0.427), *p* = 0.022	r = 0.309 (95% CI: 0.109 to 0.485), *p* = 0.003	r = 0.448 (95% CI: 0.266 to 0.599), *p* < 0.001	r = 0.665 (95% CI: 0.531 to 0.767), *p* < 0.001	—	
Distance from Labrale inferius to E-line	r = −0.204 (95% CI: −0.394 to 0.003), *p* = 0.054	r = 0.126 (95% CI: −0.083 to 0.325), *p* = 0.235	r = 0.217 (95% CI: 0.010 to 0.406), *p* = 0.040	r = 0.261 (95% CI: 0.057 to 0.444), *p* = 0.013	r = 0.490 (95% CI: 0.315 to 0.633), *p* < 0.001	r = 0.601 (95% CI: 0.450 to 0.719), *p* < 0.001	—

**Table 4:** Correlations among facial angles.

	Nasofrontal Angle	Nasolabial Angle	Mentolabial Angle	Facial Angle
Nasofrontal angle	—			
Nasolabial angle	r = −0.001 (95% CI: −0.208 to 0.206), *p* = 0.990	—		
Mentolabial angle	r = 0.023 (95% CI: −0.185 to 0.229), *p* = 0.827	r = 0.360 (95% CI: 0.165 to 0.528), *p* < 0.001	—	
Facial angle	r = −0.035 (95% CI: −0.240 to 0.173), *p* = 0.745	r = 0.385 (95% CI: 0.193 to 0.548), *p* < 0.001	r = −0.391 (95% CI: −0.553 to −0.200), *p* < 0.001	—

**Table 5:** Comparison of facial dimensions and angles by gender (N = 90).

Characteristic	Female (n = 57, 63.3%)	Male (n = 33, 36.7%)	Total (n = 90)	*p*-Value
Age, years	22.0 (12.0–27.0)	11.0 (9.0–16.0)	14.0 (10.0–24.0)	0.002
Upper facial height, mm	57.2 (54.5–59.4)	55.4 (47.9–59.0)	56.2 (53.1–59.3)	0.105
Middle facial height, mm	57.1 (53.2–60.8)	53.5 (49.0–58.0)	55.7 (52.0–60.1)	0.002
Lower facial height, mm	60.3 (56.3–63.5)	59.2 (53.7–65.5)	60.2 (55.6–63.9)	0.940
Vertical facial proportion I	1.0 (1.0–1.0)	1.0 (0.9–1.1)	1.0 (0.9–1.1)	0.335
Vertical facial proportion II	1.0 (1.0–1.1)	1.1 (1.0–1.2)	1.1 (1.0–1.1)	0.067
Total facial height, mm	175.2 (167.6–180.1)	165.4 (152.9–179.4)	173.0 (162.2–179.9)	0.045
Upper lip length, mm	15.5 (13.7–17.6)	16.2 (14.6–18.6)	15.9 (13.8–18.0)	0.175
Lower lip length, mm	38.0 (35.1–41.1)	36.6 (32.1–42.8)	37.1 (33.2–41.2)	0.368
Interpupillary distance, mm	59.1 (56.1–62.1)	58.6 (55.0–61.7)	58.8 (55.4–61.7)	0.506
Bizygomatic width, mm	110.3 (105.2–115.5)	107.8 (101.9–112.9)	109.8 (104.5–115.1)	0.187
Nasofrontal angle, °	144.6 (141.2–150.0)	142.1 (138.6–146.4)	144.1 (140.4–147.6)	0.025
Nasolabial angle, °	114.0 (107.0–120.2)	122.1 (115.2–125.2)	116.5 (108.5–123.0)	0.001
Mouth width, mm	52.0 (47.4–56.3)	49.7 (43.9–54.1)	51.0 (46.5–55.7)	0.152
Mentolabial angle, °	136.6 (124.7–145.4)	139.9 (130.5–150.0)	137.5 (125.9–148.5)	0.171
Facial angle, °	13.7 (9.5–18.5)	15.0 (10.1–20.8)	14.2 (10.0–19.5)	0.288
Distance from Labrale superius to E-line, mm	4.5 (2.1–7.1)	2.8 (1.1–4.9)	3.8 (1.6–6.6)	0.041
Distance from Labrale inferius to E-line, mm	2.6 (1.4–4.8)	2.5 (1.3–3.6)	2.6 (1.3–4.5)	0.269

Footer: IQR = interquartile range; E-line = esthetic line; mm = millimeters; ° = degrees. *p*-values obtained by two-tailed Mann–Whitney U-tests. Note: “Vertical Facial Proportion I” and “Vertical Facial Proportion II” represent two distinct proportional indices calculated from different facial height segment ratios.

## Data Availability

All data related to this manuscript can be requested from the corresponding author at alexandru.motofelea@umft.ro.
